# Synthesis and Adrenolytic Activity of New Propanolamines

**DOI:** 10.3390/molecules15063887

**Published:** 2010-05-28

**Authors:** Grażyna Groszek, Agata Bajek, Agnieszka Bis, Agnieszka Nowak-Król, Marek Bednarski, Agata Siwek, Barbara Filipek

**Affiliations:** 1 Faculty of Chemistry, Rzeszów University of Technology, 6 Powstańców Warszawy Avenue, 35-959 Rzeszów, Poland; 2 Laboratory of Pharmacological Screening, Jagiellonian University Medical College, 9 Medyczna, 30-689 Kraków, Poland; 3 Department of Pharmacobiology, Jagiellonian University Medical College, 9 Medyczna, 30-689 Kraków, Poland

**Keywords:** α_1_-andrenoceptor antagonist, synthesis, pharmacology

## Abstract

The synthesis of (2*R,S*)-1-(6-methoxy-4-(methoxymethyl)-1*H*-indol-5-yloxy)-3-(2-(2-methoxyphenoxy)ethylamino)propan-2-ol and (2*R,S*)-1-(4-methoxy-6-(methoxy­methyl)-1*H*-indol-5-yloxy)-3-(2-(2-methoxyphenoxy)ethylamino)propan-2-ol is described. The compounds were tested for electrographic, antiarrhythmic, hypotensive, and spasmolytic activity, as well as for α_1_-, α_2_- and β_1_-adrenoceptor binding affinity.

## 1. Introduction

**Figure 1:**
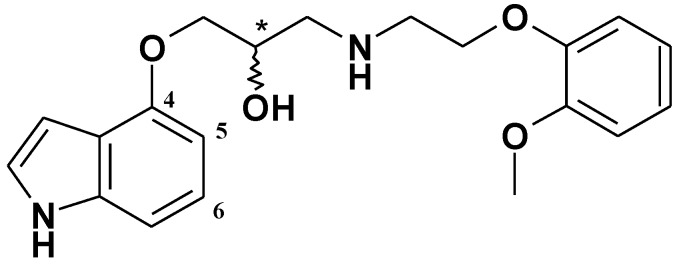
Chemical structure of the compound **(*R,S*)-9**.

In our search for new aminopropan-2-ol derivatives with cardiovascular activity among, we obtained the compound (2*R,S*)-1-(1*H*-indol-4-yloxy)-3-(2-(2-methoxyphenoxy)ethylamino)propan-2-ol, **(*R,S*)-9 **(Figure 1) [1] which became a lead structure for further investigations. The compound **(*R,S*)-9** possesses α_1_- and β_1_-adrenolytic, antiarrhythmic, and hypotensive activities similar to carvedilol, which is a very effective compound in the treatment of such cardiovascular diseases as hypertension, heart failure, and stable angina pectoris. Carvedilol also decreases secondary cardiac events after myocardial infarction (MI) and reduces infarct size after myocardial ischemic and reperfusion injury [2].

Several adrenergic drive indices, such as plasma norepinephrine, norepinephrine spillover from adrenergic nerve terminals and efferent postganglionic muscle sympathetic nerve traffic, have all shown an increase in the different conditions clustering in metabolic syndrome, such as obesity, hypertension and insulin resistance state [3]. The effects of catecholamines are of clinical significance because they lead to the final common pathway underlying cardiovascular events atherogenesis. The blockade of the α_1_-AR may result in an improvement in the metabolic profile of the diabetic hypertensive patient, as recently seen in the GEMINI trial. Catecholamines also decrease LDL receptors and lipoprotein lipase activity, and inhibit extrahepatic cholesterol synthesis so modulation of catecholamine activity may have an important role in determining the level of cholesterol [4].

β-Blockers, nonselective and β_1_ selective may promote atherogenic lipid changes as well as increased insulin resistance and glucose and insulin blood levels; β-blocker use has been associated with the degradation of glycemic control in diabetic patients and an increase in the rate of new-onset diabetes in nondiabetic patients. In contrast, carvedilol use has been associated with a beneficial or neutral effect on blood lipid levels and insulin sensitivity [5].

**Figure 2:**
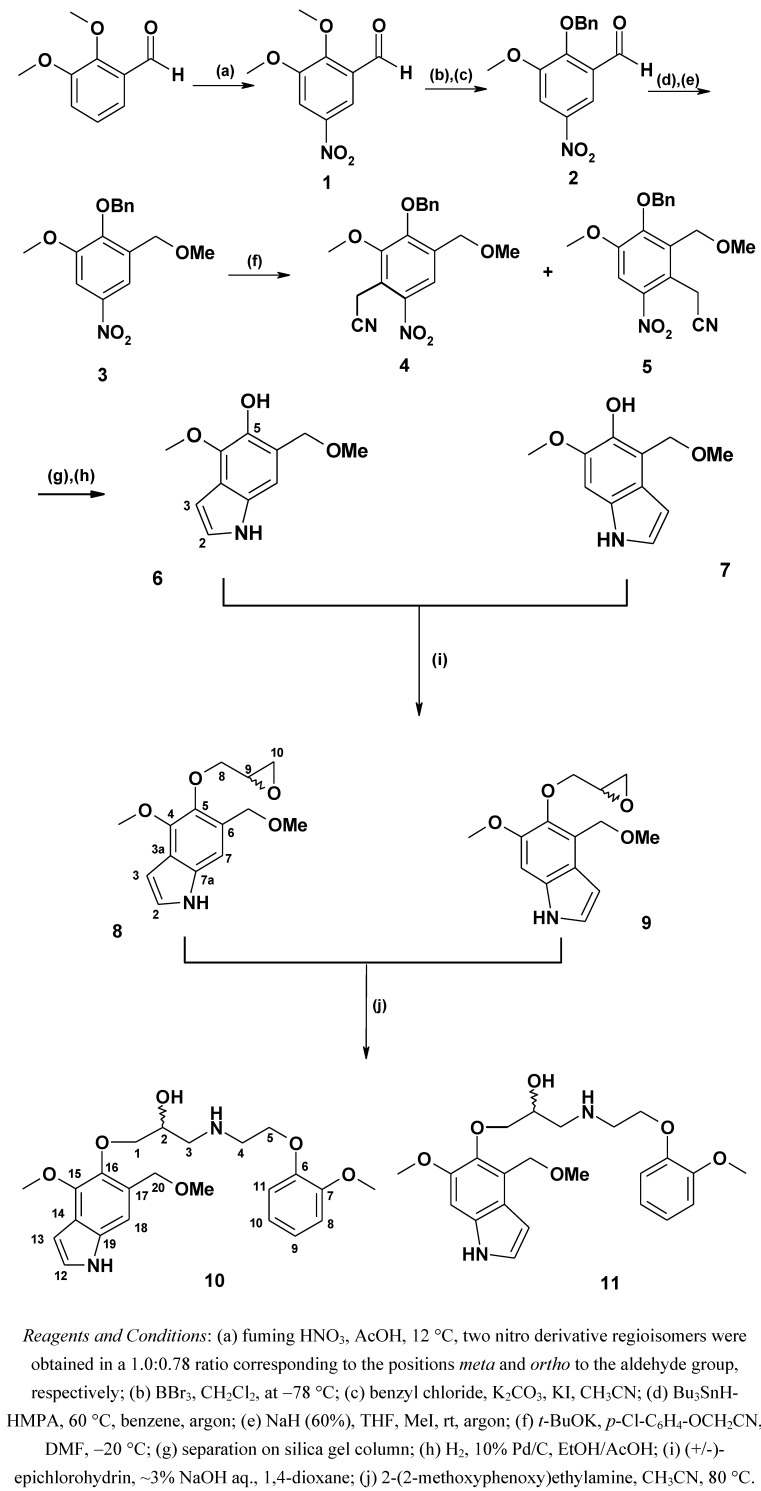
Synthetic pathway.

The aim of this study was the synthesis and evaluation of the cardiovascular activity of two new aminopropan-2-ol derivatives: (2*R,S*)-1-(6-methoxy-4-(methoxymethyl)-1*H*-indol-5-yloxy)-3-(2-(2-methoxyphenoxy)ethylamino)propan-2-ol and (2*R,S*)-1-(4-methoxy-6-(methoxymethyl)-1*H*-indol-5-yloxy)-3-(2-(2-methoxyphenoxy)ethylamino)propan-2-ol (Figure 2). The tested compounds are structural analogs of compound **(*R,S*)-9**, and other compounds described in the previous articles [6]. However here, aminopropanol moiety is situated in the 5-position of the indole moiety and what is more, the indole ring contains two different substituents, methoxy- and methoxymethyl.

## 2. Results and Discussion

### 2.1. Chemistry

The synthesis of target compounds is outlined in Figure 2. Commercially available 2,3-dimethoxybenzaldehyde was nitrated using fuming nitric acid in acetic acid to produce two nitrobenzaldehyde derivatives – the 5-nitro-isomer **1** and its regioisomer, 2,3-dimethoxy-6-nitrobenzaldehyde in a 1:0.78 ratio, respectively. They were readily separated by column chromatography on silica gel. The latter was used in another project to obtain an indole derivative [6]. Selective monodemethylation of compound **1** with BBr_3_ followed by benzylation of the phenolic hydroxyl group led to **2** in 75% yield. Reduction of the aldehyde derivative **2** was achieved with the combined Bu_3_SnH-HMPA system as described by Shibata *et al.* [7]. The method is suitable for benzaldehyde derivatives bearing other reducible functional groups such as nitro. Attempts to utilize the obtained benzyl alcohol derivative for further reaction such as *O*-methylation in a one pot procedure have failed. In our case it turned out that standard work-up had to be done after reduction of aldehyde and before the alkylation step. Then, *O*-alkylation became possible and was performed using NaH and MeI in THF at 5 °C to room temperature. Compound **3** was obtained in a yield of 78%. Vicarious nucleophilic substitution of hydrogen [8] in the nitrobenzene derivative **3** with (4-chlorophenoxy)acetonitrile in the presence of potassium *t*-butoxide in DMF gave two regioisomers **4** and **5**, containing the cyanomethyl substituent in positions *ortho* to the nitro group. They were separated *via* column chromatography on silica gel. For intermediates **4** and **5**, the yields were 29% and 43%, respectively. Both isomers were converted to the hydroxyindole derivatives **6** and **7** in 67% yield by means of reductive cyclization of the corresponding cyanomethyl derivatives. In the ^1^H-NMR spectrum of compounds **6** and **7**, the aromatic proton in position 3- appeared as multiplets at 6.47–6.48, and 6.46–6.47 ppm, respectively. The proton in position 2- appeared as an apparent triplet at 7.21 ppm (*J* 2.8) for the product **6. **On the contrary, this proton in product **7** appeared as a doublet of doublets at 7.12 ppm (*J* 3.1 and 2.5). The phenolic hydroxyl was situated in position 5- of the indole framework. As described in the previous paper [1], compounds **6** and **7** were transformed by condensation with (+/-)-epichlorohydrin. The epoxide derivatives **8** and **9** were formed and then converted to the aminoalcohols **10** and **11** by addition reaction of 2-(2-methoxyphenoxy)ethylamine to the epoxide function. The epoxides **8** and **9** were obtained in yields of 44% to 64%, and the aminoalcohol derivatives **10** and **11** in yields of 70% and 68%, respectively.

### 2.2. Pharmacology

#### 2.2.1. Radioligand receptor binding assay for α- and β-adrenergic receptors

The affinity of compounds **10 **and **11 **to the catecholamine binding side of α_1_-, α_2_- and β_1_-adrenoceptors was measured as a rate of specific displacement of [^3^H]Prazosin, [^3^H]Clonidine and [^3^H]CGP12177 at the concentrations of 0.2 nM, 2 nM and 0.2 nM, respectively. Compound **11** displaced [^3^H]Prazosin from cortical binding sites in higher concentration than compound **10** (K_i_ = 21.4 nM and 5,9 nM, respectively) and inhibited [^3^H]Clonidine in lower concentration than compound **10** (K_i_ = 166.5 nM and 306.7 nM, respectively). These compounds displaced [^3^H]CGP12177 from its binding sites in low concentration rate (K_i_ = 4.2–6.9 μM). The results are summarized in Table 1.

**Table 1:** Affinity for different adrenoceptor types in the rat cerebral cortex (K_i_ [nM] ± SEM).

Compound	[^3^H]Prazosin	[^3^H]Clonidine	[^3^H]CGP12177
**10**	5.9 ± 0.3	306.7 ± 11.7	6.9 ± 0,8 μM
**11**	21.4 ± 1.3	166.5 ± 9.8	4.2 ± 0,6 μM
**(*R,S*)-9**	89.8 ± 9.5	1.4 ± 0.4 μM	3.0 ± 0.6
Carvedilol	2.2 ± 0.2^a^	3.4 ± 0.9 μM	0.81 ± 0.06^a^

^a^ Ref. [9].

None of the new analogs of compound **(*R,S*)-9** displaced [^3^H]CGP12177 from the cortical binding site at low concentration. Both of them had high affinity to α-adrenoceptors and compound **10** was 52-fold more selective towards α_1_-adrenergic receptors than compound **11**. In the case of α_2_-adrenoceptors, **11** possessed twice as much affinity as **10**. The affinity to specific receptors may suggest the direction of biological action of the new tested compounds.

#### 2.2.2. Effect on normal electrocardiogram (ECG) *in vivo* in rats

The effects on ECG intervals and heart rate were determined for all tested compounds at the same dose of 1 mg∙kg^-1^. The influence of compound **10** on the ECG was similar to that observed after administration of compound **11**. The tested compounds significantly changed the ECG pattern. They prolonged P–Q, Q–T intervals, and QRS complex. The compound **11** slightly decreased the number of cardiac beats per minute, up to 15 min after administration (Table 2).

**Table 2:** Effects of an *iv* injection of the investigated compounds in dose of 1 mg∙kg^-1^ on heart rate and ECG intervals in anesthetized male Wistar rats (60 mg of thiopental∙kg^-1^, *ip*).

Compound Parameters				Time of observation (min)								
				0		1		5		15		
**10**		P–Q (ms)		40.4 ± 0.4		45.6 ± 1.6^b^		46.8 ± 1.6^c^		46.4 ± 1.6^c^		
		QRS (ms)		27.6 ± 0.8		31.2 ± 0.5^c^		31.2 ± 1.0^c^		32.4 ± 0.8^d^		
		Q–T (ms)		94.4 ± 2.8		92.0 ± 4.2		100.0 ± 2.8		104.0 ± 1.4^a^		
		Beats/min		329.4 ± 12.2		334.0 ± 13.0		309.6 ± 13.7		297.4 ± 12.4		
**11**		P–Q (ms)		42.8 ± 1.2		47.2 ± 1.0^c^		46.8 ± 0.5^b^		48.0 ± 1.1^c^	
		QRS (ms)		27.2 ± 1.4		30.4 ± 0.4^b^		31.2 ± 0.8^c^		32.4 ± 0.8^d^	
		Q–T (ms)		85.2 ± 0.8		87.2 ± 1.2		91.2 ± 3.3		97.2 ± 2.2^d^	
		Beats/min		337.8 ± 6.7		332.4 ± 10.6		320.6 ± 7.8		310.2 ± 9.4^a^	
**(*R,S*)-9**		P–Q (ms)		45.7 ± 2.0		47.2 ± 1.6		48.1 -± 2.4		46.2 ± 1.7	
		QRS (ms)		26.4 ± 1.3		28.4 ± 1.2		24.9 ± 1.3		26.9 ± 1.1	
		Q–T (ms)		72.6 ± 1.7		69.2 ± 1.1		69.0 ± 1.6		72.8 ± 1.3	
		Beats/min		304.9 ± 8.9		298.7 ± 7.2		294.5 ± 7.7		287.3 ± 7.9	
Carvedilol		P–Q (ms)		50.0 ± 3.2		50.0 ± 3.2		55.0 ± 5.5		54.6 ± 3.0	
		QRS (ms)		21.2 ± 0.8		22 ± 0.6		22.8 ± 1.0		23.2 ± 0.8	
		Q–T (ms)		72.0 ± 3.1		74.4 ± 2.8		68.0 ± 3.7		74.0 ± 2.4	
		Beats/min		356.7 ± 21.0		345.2 ± 17.7		340.3 ± 14.8		320.4 ± 10.2	

Values are the mean ± SEM of 6 experiments; The statistical analyses were performed using a one-way ANOVA test; ^a^ p < 0.05; ^b^ p < 0.02; ^c^ p < 0.01; ^d^ p < 0.001.

The electrocardiographic changes observed after administration of these compounds were similar to those seen after administration of quinidine [10]. These quinidine-like properties also known as membrane stabilizing activities could partially cause the antiarrhythmic activities of the tested compounds.

#### 2.2.3. Effect on adrenaline-induced arrhythmia in rats

The prophylactic antiarrhythmic activity of compounds **10** and **11 **was determined using an adrenaline-induced arrhythmia model of in anesthetized rats. Intravenous injections of adrenaline at a dose of 20 μg∙kg^-1 ^caused reflex bradycardia (100%), supraventricular and ventricular extrasystoles (100%), bigeminy in rats, which led to the death of approximately 50% of the test animals. The ED_50_ values (a dose producing 50% inhibition of premature ventricular beats) are presented in Table 3.

**Table 3:** The prophylactic antiarrhythmic activity in anesthetized rats.

Compound	ED_50_ *iv* (mg∙kg^-1^)	ED_50_ *po* (mg∙kg^-1^)
**10**	0.35 (0.18–0.72)	1.71 (1.52 ± 1.93)
**11**	0.16 (0.10–0.23)	0.86 (0.76 ± 0.96)
**(*R,S*)-9**	0.34 (0.23–0.51)	0.44 (0.18 ± 1.10)
Carvedilol	0.25 (0.12–0.53)	−
Propranolol	1.05 (0.64–1.73)^a^	19.5 (14.5 ± 26.1)

^a^ Ref. [11].

The tested compounds injected intravenously 15 min before adrenaline, prevented and/or diminished the number of premature ventricular and supraventricular beats, and reduced mortality. The compound **11 **was more active, exhibited important antiarrhythmic effects, and its ED_50 _value equaled 0.16 mg∙kg^-1^. The ED_50_ value for compound **11 **was about two times lower than that for compound **10**.

These compounds administered orally (intragastric) 60 min before adrenaline retained (maintained) antiarrhythmic activity. The ED_50_ value equaled 0.86 mg∙kg^-1^ and 1.71 mg∙kg^-1^, for compounds **11 **and **10**, respectively. In cardiac myocytes, stimulation of β-adrenergic receptors increased the magnitude of I_Ca-L_ (Ca^2+^ current) *via* protein kinase A-dependent phosphorylation of the following channels: I_f_ (an inward current carried by Na^+^ and K^+^), I_Cl_ (induced Cl^–^ current), I_Kur_ (an ultrarapid delayed rectifier K^+^ current), I_KATP_ (an ATP-independent K^+^ current), and I_st_, these actions being mediated by cAMP dependent protein kinase [12,13,14]. The β-adrenergic stimulation produced by voltage-dependent Ca^2+^, I_Kur_ as well as Na^+^ channels increased the probability of occurrence of a variety of supraventricular and ventricular arrhythmias [15]. The stimulation of α_1_-adrenergic receptors inhibited the α-adrenergic activation of the Cl^–^ current [12]. The tested compounds given *iv* 15 min before arrhythmogen prevented or attenuated the symptoms of adrenaline-induced arrhythmia. The antiarrhythmic activity of the tested compounds is lower than the effect of carvedilol and compound **(*R,S*)-9**, and it seems to be related to their adrenolytic properties. These results are in good agreement with previously published data on non-selective and selective α-antagonists such as phentolamine, prazosin, and abanoquil which prevented arrhythmia induced by adrenaline or cocaine infusions [16,17]. The obtained results have shown, despite low β-adrenolytic activities, the antiarrhythmic effect of compounds **10** and **11**.

#### 2.2.4. Influence on blood pressure in rats

Hypotensive activity of compounds **10** and **11** was determined after *iv* and *po* administration to normotensive anesthetized rats. Compound **11** significantly decreased the systolic (9–20%) and diastolic (9–20%) blood pressure throughout the whole observation period in the range of doses 0.125–1 mg∙kg^-1^ after *iv* administration. 

**Figure 3:**
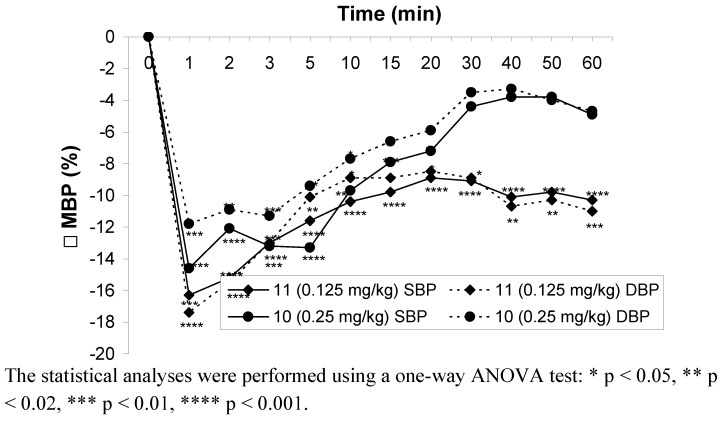
Changes in mean blood pressure after *iv* administration of the tested compounds in the lowest effective doses.

At the lowest dose (0.0625 mg·kg^-1^) the hypotensive activity of compound **11** disappeared. Compound **10 **at a dose of 0.5 mg∙kg^-1 ^significantly decreased systolic blood pressures (8–14%) throughout the whole observation period and diastolic blood pressure only at the first 10 min after *iv* administration. At the lower dose (0.25 mg·kg^-1^) this compound decreased systolic and diastolic blood pressure in 20 and 10 min after administration, respectively (Figure 3).

After *po* administration the compound **11** in the dose of 0.5 mg·kg^-1 ^significantly decreased systolic (12–16%) and diastolic (10–13%) blood pressures from 30 to 70 min and from 30 to 60 min, respectively. In the lower dose (0.25 mg∙kg^-1^) this compound decreased systolic and diastolic blood pressures from 30 to 90 min, and from 40 to 90 min after administration, respectively. Hypotensive activity of compound **11 **disappeared in the dose of 0.125 mg·kg^-1^.

**Figure 4:**
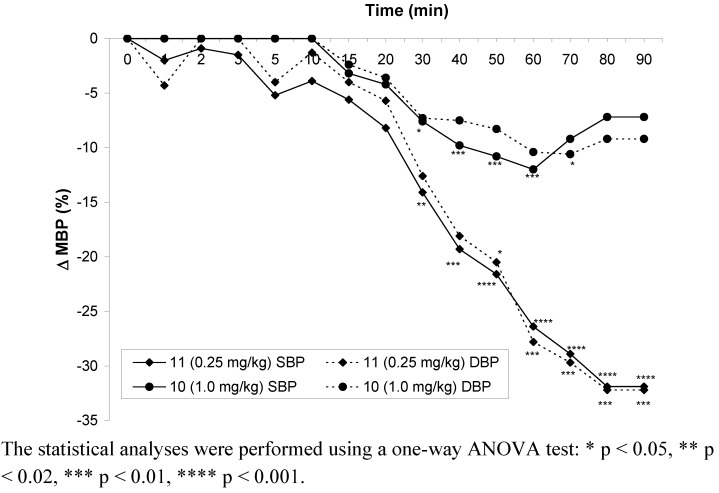
Changes in mean blood pressure after *po* administration of the tested compounds in the lowest effective doses.

Compound **10 **significantly decreased systolic (9–18%) and diastolic (11–23%) blood pressures in the dose of 2 mg∙kg^-1^ from 40 to 90 min after *po* administration (to the end of the observation period). The hypotensive activity disappeared in the dose of 1 mg∙kg^-1^ of compound **10 **(Figure 4).

Both tested compounds have revealed hypotensive activity after *iv* and *po* administration to normotensive rats. Compounds **10** and **11** significantly decreased blood pressure throughout the whole observation period at doses of 0.125 mg∙kg^-1^ and 0.5 mg∙kg^-1^ after *iv* administration. A dose four times larger administered orally maintained the some level of hypotensive action as when administered intravenously. The α_1_-adrenoreceptor blockade in blood vessels is probably caused by hypotensive activity. Classic α_1_-antagonist prazosins proved to be a highly effective antihypertensive drug, and its congeners, doxazosin and terazosin, which offer similar pharmacology with a longer duration of action, remain important options in the treatment regimen available for hypertension. In addition to blood pressure reduction, α_1_-antagonists offer the advantages of a favourable effect on plasma lipoproteins and a low incidence of sexual dysfunction [10,18,19]. α_1_-Adrenergic receptor blockers can be used for the initial treatment of hypertension (and may be preferred in patients with BPH, that is, benign prostatic hypertrophy). They can improve voiding symptoms such as hesitancy, straining, and weak urine flow [18,19].

#### 2.2.5. Influence on isolated rabbit ileum

Compound **10** statistically significantly diminished amplitude of contractions of isolated rabbit small intestine in the range of concentrations 10^-8^–10^-5^ M from 14 to 35%. At the highest concentration (10^-5^ M) the tested compound significantly increased frequency of contractions of isolated rabbit small intestine of about 8% (Table 4).

The compound **11**, only given at the highest concentration of 10^-5^ M statistically significantly decreased the amplitude of contractions of isolated rabbit small intestine of 30%, but none of the tested doses of compound **11 **influenced the frequency of contractions of isolated rabbit small intestine.

The spasmolytic activity of the tested compounds could confirm that hypotensive activity is caused by α-adrenolytic activity and additional spasmolytic one.

**Table 4:** The prophylactic antiarrhythmic activity in anesthetized rats.

Compounds\Concentration		0	10^-9 ^[M]	0	10^-8^ [M]	0	10^-7 ^[M]	0	10^-6 ^[M]	0	10^-5 ^[M]
**Amplitude [g]**	**10**	1.43 ± 0.06	1.56 ± 0.06	1.53 ± 0.06	1.31 ± 0.06^b^	1.55 ± 0.10	1.29 ± 0.05^a^	1.63 ± 0.10	1.10 ± 0.07^c^	1.72 ± 0.11	1.11 ± 0.09^c^
	**11**	2.54 ± 0.28	2.59 ± 0.22	1.33 ± 0.09	1.68 ± 0.13^a^	2.06 ± 0.21	166 ± 0.13	2.15 ± 0.12	2.02 ± 0.11	2.62 ± 0.19	1.82 ± 0.13^b^
**Frequency [min ^-1^]**	**10**	12.74 ± 0.20	12.14 ± 0.22	11.77 ± 0.14	12.15 ± 0.18	12.05 ± 0.19	12.15 ± 0.15	12.24 ± 0.25	11.60 ± 0.22	11.61 ± 0.27	10.70 ± 0.25^a^
	**11**	6.90 ± 0.12	8.23 ± 0.95	11.48 ± 1.29	9.97 ± 1.13	10.44 ± 1.19	10.95 ± 1.19	12.08 ± 1.02	13.59 ± 0.67	11.40 ± 1.17	8.88 ± 0.85

Values are the mean ± SEM of 4-5 experiments; The statistical analyses were performed using a one-way ANOVA test; ^a^ p < 0.05; ^b^ p < 0.01; ^c^ p < 0.001.

## 3. Experimental

### 3.1. Chemistry

#### 3.1.1. General

Melting points were determined on a Boëtius apparatus and have gone uncorrected. ^1^H- and ^13^C- NMR spectra were recorded on a Bruker (500 MHz) instrument. Chemical shifts are expressed in ppm (δ) referred to TMS, coupling constants (*J*) are given in Hz. IR and UV spectra were recorded on Perkin-Elmer and Hewlett Packard 8453 instruments, respectively. IR spectra were recorded in KBr pallets and wavenumber expressed in cm^-1^. Elemental analysis was done on AE 1108 Carlo Erba apparatus. Mass spectra were obtained on AMD-604 spectrometer. TLC plates precoated with silica gel 60 F_254_ were used for monitoring, and silica gel 230–400 mesh was used for flash column chromatography (both from Merck).

#### 3.1.2. Materials

Sodium hydride (60% in mineral oil) was supplied by Avocado Res. Chem. Ltd. (+/-)-2-(Chloromethyl)oxirane was purchased from Aldrich Chemicals. Fuming nitric acid (d = 1.52), tributyltin hydride, 2,3-dimethoxybenzaldehyde, boron tribromide solution 1.0 M in DCM, were obtained from Fluka Chemicals. Solvents were distilled and dried if required. Other common materials were commercial products. 2-(2-Methoxyphenoxy)ethylamine and (4-chlorophenoxy)acetonitrile were obtained according to literature procedures [1,20,21]. [^3^H]Prazosin, [^3^H]Clonidine and [^3^H]CGP12177 were supplied by Perkin−Elmer. The reference compound carvedilol was obtained from the Pharmaceutical Research Institute, Warsaw, Poland.

#### 3.1.3. *2,3-Dimethoxy-5-nitrobenzaldehyde* (**1**)

2,3-Dimethoxybenzaldehyde was converted to 2,3-dimethoxy-5-nitrobenzaldehyde (1) with a yield of 46% by the method described by Fukuyama *et al*. [22]. The regioisomer 2,3-dimethoxy-6-nitrobenzaldehyde was also obtained with a yield of 36% [6]. Data for compound **1**: Mp 117*–*118 °C (AcOEt); IR: 743.3, 979.3, 1246.5, 1280.1, 1336.2, 1484.2, 1520.5, 1585.4, 1687.5 (C=O) and 3094.0 cm^-1^; UV (EtOH, c = 0.064 mg/10 mL; nm), λ_max_: 211 (lgε = 4.23) and 269 (lgε = 4.34); ^1^H-NMR (CDCl_3_): δ 4.02 and 4.15 (2xs, 6H, 2xOC*H*_3_), 7.96 and 8.30 (2xd, *J* 2.7, 2H, Ar*H*), 10.42 (s, 1H, C*H*O); ^13^C-NMR (CDCl_3_): δ 56.6 (O*C*H_3_), 62.5 (O*C*H_3_), 111.7, 115.3, 128.8, 143.5, 153.2, 157.2 and 188.1 (*C*=O); MS (EI, 70 eV), m/z [%]: 211 (100, M^+^), 193 (69), 182 (16), 165 (43), 150 (49), 135 (38), 121 (31), 107 (28), 79 (17), 51 (19); HR-MS for C_9_H_9_NO_5_; calcd. 211.0481, found: 211.0486.

#### 3.1.4. *2-(Benzyloxy)-3-methoxy-5-nitrobenzaldehyde* (**2**)

2,3-Dimethoxy-5-nitrobenzaldehyde (**1**) was subjected to regioselective deprotection of the hydroxyl group according to the literature [22], and 2-hydroxy-3-methoxy-5-nitrobenzaldehyde was produced with a yield of 98%, mp 139–141 °C (DCM/hexane); IR: 766.2, 957.0, 1087.2, 1099.9, 1205.2, 1267.0, 1345.1, 1464.5, 1530.9, 1654.2 (C=O), 3080.0 and 3294.9 (OH) cm^-1^; UV (EtOH, c = 0.0796 mg/10 mL; nm), λ_max_: 262 (lgε = 3.93), 373 (lgε = 4.03) and 419 (lgε = 4.01); ^1^H-NMR (CDCl_3_): δ 4.03 (s, 3H, OC*H*_3_), 7.94 (d, *J* 2.5, 1H, Ar*H*), 8.23 (d, *J* 2.5, 1H, Ar*H*), 10.01 (s, 1H, C*H*O), 11.72 (s, 1H, O*H*); ^13^C-NMR (CDCl_3_): δ 56.8 (O*C*H_3_), 111.4, 118.8, 120.5, 140.4, 149.1, 156.9, 195.5 (*C*HO); MS (EI, 70 eV), m/z [%]: 197 (100, M^+^), 179 (10), 151 (65), 136 (35), 121 (12), 108 (39), 80 (25), 63 (11), 51 (22); HR-MS for C_8_H_7_NO_5_; calcd. 197.0324, found: 197.0321; Anal. calcd. for C_8_H_7_NO_5_: C–48.74, H–3.58, N–7.10, found: C–48.69, H–3.59, N–6.98.

The latter was converted into product **2** according to a procedure described in literature [6]. The crude product was crystallized from DCM/hexane to give compound **2 **(yield 77%)as a colorless crystalline solid with mp 123–124 ºC; IR: 744.5, 957.9, 1093.0, 1235.3, 1337.9, 1344.2, 1525.5 (C=C), 1691.7 (C=O) and 3099.7 cm^-1^; UV (EtOH, c = 0.088 mg/10 mL; nm), λ_max_: 212 (lgε = 4.36) and 243 (lgε = 4.12); ^1^H-NMR (CDCl_3_): δ 4.06 (s, 3H, OC*H*_3_), 5.35 (s, 2H, C*H*_2_), 7.33–7.38 (m, 5H, Ar*H*), 7.97 (d, *J* 2.7, 1H, Ar*H*), 8.25 (d, *J* 2.7, 1H, Ar*H*) 10.17 (s, 1H, C*H*O); ^13^C-NMR (CDCl_3_): δ 56.7 (O*C*H_3_), 76.6 (*C*H_2_), 111.5, 115.1, 128.7, 128.8, 129.1, 129.6, 135.4, 143.8, 153.4, 155.6 and 188.1 (*C*=O); MS (EI, 70 eV), m/z [%]: 287 (<1, M^+^), 258 (1*),* 175 (16), 132 (9), 91 (100), 65 (10), 40 (12);HR-MS for C_15_H_13_NO_5_; calcd. 287.0794, found: 287.0783; Anal. calcd. for C_15_H_13_NO_5_: C–62.72, H–4.56, N–4.88; found: C–62.95, H–4.66, N–4.94.

#### 3.1.5. *2-(Benzyloxy)-1-methoxy-3-(methoxymethyl)-5-nitrobenzene* (**3**)

The nitrobenzaldehyde **2 **(10.05 g; 0.035 mol) dissolved in dry benzene (40 mL), was added dropwise to the stirred mixture of Bu_3_SnH (18.5 mL; 0.07 mol) and HMPA (12.5 mL; 0.07 mol) under an argon atmosphere. The reaction mixture was stirred at 60 °C for 3 h until disappearance of substrate (monitoring by TLC). Methanol (20 mL) was added to the reaction mixture which was then concentrated (rotary evaporator). The crude product was purified by column chromatography (silica gel, eluted with hexane/AcOEt; 3:2), and crystallized from AcOEt/petroleum ether. (2-Benzyloxy-3-methoxy-5-nitrophenyl)methanol (8.7 g; 82%) was obtained as a brightly-yellow crystalline solid with mp 67–70 ºC; IR: 1036.9, 1045.9, 1350.0, 1478.1, 1522.1, 3269.2 (OH) cm^-1^; UV (EtOH, c = 0.12 mg/10 mL; nm), λ_max_: 213 (lgε = 4.32); ^1^H-NMR (CDCl_3_): δ 1.93 (brs, 1H, O*H*), 3.99 (s, 3H, OC*H*_3_), 4.56 (s, 2H, C*H*_2_OH), 5.20 (s, 2H, C*H*_2_), 7.33–7.39 (m, 5H, Ar*H*), 7.77 (d, *J* 2.7, 1H, Ar*H*), 7.91 (d, *J* 2.7, 1H, Ar*H*); ^13^C-NMR (CDCl_3_): δ 56.3 (O*C*H_3_), 60.6 (*C*H_2_OH), 75.3 (*C*H_2_), 107.1, 116.2, 128.5, 128.7, 135.5, 136.6, 143.7, 150.5 and 152.2; MS (EI, 70 eV), m/z [%]: 289 (<1, M^+^), 171 (2), 91 (100), 65 (8), 40 (6); HR-MS for C_15_H_15_NO_5_; calcd. 289.0950, found: 289.0956; Anal. calcd. for C_15_H_15_NO_5_: C–62.28, H–5.23, N–4.84; found: C–62.32, H–5.21, N–4.85.

(2-Benzyloxy-3-methoxy-5-nitrophenyl)methanol (8.32 g; 29 mmol) was dissolved in THF (55 mL) and cooled to 5 ºC (ice/water bath). Sodium hydride (60% in mineral oil; 1.56 g; 39 mmol) was added followed by methyl iodide (6 mL; 97 mmol). The reaction mixture was allowed to warm to room temperature and kept for 1 h. Then it was poured into ice cooled water (300 mL). The product was extracted with DCM (2 x 150 mL). The combined organic layer were washed with water and brine, and then dried over anhydrous magnesium sulphate. After solvent removal (to dryness), the crude product was obtained as oil. The product was passed through a chromatographic column (silica gel, eluent hexane/AcOEt), and crystallized from acetone/petrol ether to give ether **3 **(8.3 g; 95%) as a colorless crystalline solid, mp 59–60.5 ºC; IR: 700.3, 954.1, 1069.5, 1093.6, 1342.6, 1477.0 and 1525.4 cm^-1^; UV (EtOH, c = 0.107 mg/10 mL; nm), λ_max_: 213 (lgε = 4.34); ^1^H-NMR (CDCl_3_): δ 3.35 (s, 3H, CH_2_OC*H*_3_), 3.98 (s, 3H, OC*H*_3_), 4.38 (s, 2H, C*H*_2_OCH_3_), 5.15 (s, 2H, C*H*_2_), 7.33–7.41 (m, 5H, Ar*H*), 7.76 (d, *J* 2.7, 1H, Ar*H*), 7.95 (d, *J* 2.7, 1H, Ar*H*); ^13^C-NMR (CDCl_3_): δ 56.3 (O*C*H_3_), 58.6 (CH_2_O*C*H_3_), 68.9 (*C*H_2_OCH_3_), 75.2, 106.9, 116.5, 128.4, 128.5, 128.6, 133.4, 136.8, 143.8, 150.6 and 152.3; MS (EI, 70 eV), m/z [%]: 303 (<1, M^+^), 271 (8), 91 (100), 65 (7); HR-MS for C_16_H_17_NO_5_; calcd. 303.1107, found: 303.1114; Anal. calcd. for C_16_H_17_NO_5_: C–63.36, H–5.65, N–4.62; found: C–63.24, H–5.76, N–4.71.

#### 3.1.6. *(3-(Benzyloxy)-2-methoxy-4-(methoxymethyl)-6-nitrophenyl)acetonitrile* (**4**) and *(3-(benzyloxy)-4-methoxy-2-(methoxymethyl)-6-nitrophenyl)acetonitrile* (**5**)

Benzyl derivative **3 **was subjected to the vicarious nucleophilic substitution of hydrogen with (4-chlorophenoxy)acetonitrile in the presence of potassium *t*-butoxide in DMF according to the procedure described in the literature [23] to furnish a mixture of three products (detected on TLC) as an oil. The oily residue was chromatographed on silica gel using DCM as eluent. Three fractions, of chromatographic purity, were obtained: the first was a Thorpe condensation product [24], which was discarded; the second and third were identified as products **4** and **5**. Crystallization from ethanol gave derivative **4 **in 29% yield as colorless crystals, mp 101–103 ºC and product **5** as yellow crystals with a yield of 43%, mp 82–83 ºC.

*Product* **4**: IR: 699.4, 958.6, 1065.3, 1231.6, 1340.4, 1453.6, 1526.7, 2261.0 (CN) and 2939.7 cm^-1^; UV (EtOH, c = 0.0788 mg/10 mL; nm), λ_max_: 213 (lgε = 4.44), 285 (lgε = 3.91). ^1^H-NMR (CDCl_3_): δ 3.37 (s, 3H, CH_2_OC*H*_3_), 4.02 (s, 3H, OC*H*_3_), 4.12 (s, 2H, C*H*_2_CN), 4.35 (s, 2H, C*H*_2_OCH_3_), 5.12 (s, 2H, C*H*_2_), 7.36–7.42 (m, 5H, Ar*H*), 8.00 (s, 1H, Ar*H*); ^13^C-NMR (CDCl_3_): δ 15.3 (*C*H_2_CN), 58.8 (O*C*H_3_), 61.4 (CH_2_O*C*H_3_), 68.5 (*C*H_2_OCH_3_), 75.4 (*C*H_2_), 116.7, 120.7, 121.1, 128.4, 128.6, 128.7, 128.8, 128.9, 134.9, 135.9, 143.9, 151.8 and 153.8; MS (EI, 70 eV), m/z [%]: 342 (<1, M^+^), 321 (1), 175 (2), 91 (100), 65 (6); HR-MS for C_18_H_18_N_2_O_5_; calcd. 342.1216, found: 342.1211; Anal. calcd. for C_18_H_18_N_2_O_5_: C–63.15, H–5.30, N–8.18; found: C–63.25, H–5.42, N–8.16.

*Product* **5**: IR: 734.7, 1084.6, 1233.5, 1290.0, 1334.8, 1521.0, 1576.0, 2251.7 (CN) and 2927.7 cm^‑1^; UV (EtOH, c = 0.10 mg/10 mL; nm), λ_max_: 212 (lgε = 4.37), 246 (lgε = 4.17), 278 (lgε = 4.11); ^1^H-NMR (CDCl_3_): δ 3.30 (s, 3H, CH_2_OC*H*_3_), 3.98 (s, 3H, OC*H*_3_), 4.05 (s, 2H, C*H*_2_CN), 4.56 (s, 2H, C*H*_2_OCH_3_), 5.11 (s, 2H, C*H*_2_), 7.36–7.42 (m, 5H, Ar*H*), 7.68 (s, 1H, Ar*H*); ^13^C-NMR (CDCl_3_): δ 17.5 (*C*H_2_CN), 56.4 (O*C*H_3_), 58.4 (CH_2_O*C*H_3_), 65.2 (*C*H_2_OCH_3_), 76.3 (*C*H_2_), 109.2, 116.8, 119.6, 128.4, 128.6 (2x*C*), 128.7 (2x*C*), 132.6, 136.2, 144.9, 151.3 and 152.4; MS (EI, 70 eV), m/z [%]: 342 (3, M^+^), 91 (100), 65 (6); HR-MS for C_18_H_18_N_2_O_5_; calcd. 342.1216, found: 342.1223; Anal. calcd. for C_18_H_18_N_2_O_5_: C–63.15, H–5.30, N–8.18; found: C–63.18, H–5.32, N–8.21.

#### 3.1.7. *4-Methoxy-6-(methoxymethyl)-1H-indol-5-ol* (**6**) and *6-methoxy-4-(methoxymethyl)-1H-indol-5-ol* (**7**)

The compounds **4 **and **5** were subjected to reductive cyclization according to a procedure described in the literature [1,20]. Indole derivative **6** was obtained in 67% yield, as colorless crystals with mp 105–106 ºC (DCM/petroleum ether), and indole derivative **7 **with a yield of 67%, as colorless crystals with mp 118–118.5 ºC (DCM/petroleum ether) or mp 120–120.5 °C (acetone/petroleum ether).

Product **6**: IR: 749.0, 957.0, 1054.7, 1102.0, 1354.3, 1457.1, 1518.1, 3266.1, 3383.2 (NH), 3200-3600 (OH) cm^-1^; UV (EtOH, c = 0.0686 mg/10 mL; nm), λ_max_: 216 (lgε = 4.59) and 271 (lgε = 4.27); ^1^H-NMR (acetone-d_6_): *δ* 3.37 (s, 1H, CH_2_OC*H*_3_), 3.97 (s, 3H, OC*H*_3_), 4.57 (s, 2H, C*H*_2_OCH_3_)_,_ 6.47-6.48 (m, 1H, H-3), 7.06 (s, 1H, O*H*), 7.10 (s, 1H, Ar*H*), 7.21 (t, *J* 2.8, 1H, H-2), 10.03 (brs, 1H, N*H*); ^13^C-NMR (acetone-d_6_): *δ* 58.0 (CH_2_O*C*H_3_), 60.3 (O*C*H_3_), 71.1 (*C*H_2_OCH_3_), 98.9, 106.6, 125.4, 121.1, 122.2, 132.8, 139.0 and 140.1; MS (EI, 70 eV), m/z [%]: 207 (29, M^+^), 175 (100), 146 (39), 132 (45), 104 (16), 91 (11); HR-MS for C_11_H_13_NO_3_; calcd. 207.0895, found: 207.0905; Anal. calcd. for C_11_H_13_NO_3_: C–63.76, H–6.32, N–6.76; found: C–63.52, H–6.47, N–7.09.

Product **7**: IR: 730.8, 1074.7, 1148.3, 1311.9, 1436.8, 1471.8, 2926.8 and 3366.0 (OH) cm^-1^; UV (EtOH, c = 0.064 mg/10 mL; nm), λ_max_: 213 (lgε = 4.40) and 303 (lgε = 3.94); ^1^H-NMR (acetone-d_6_): δ 3.31 (s, 3H, CH_2_OCH_3_), 3.85 (s, 3H, OCH_3_), 4.79 (s, 2H, CH_2_OCH_3_)_,_ 6.46–6.47 (m, 1H, H-3), 6.88 (s, 1H, OH), 6.97 (s, 1H, ArH), 7.12 (dd, *J* 3.1 and 2.5, 1H, H-2), 9.84 (brs, 1H, NH); ^13^C-NMR (acetone-d_6_): δ 56.6 (OCH_3_), 57.6 (CH_2_OCH_3_), 67.5 (CH_2_OCH_3_), 101.0, 94.9, 123.7, 113.8, 122.7, 130.4, 140.6 and 145.7; MS (EI, 70 eV), m/z [%]: 207 (46, M^+^), 175 (100), 161 (13), 146 (52), 132 (80), 118 (14), 104 (34), 91 (5); HR-MS for C_11_H_13_NO_3_; calcd. 207.0895, found: 207.0886.

#### 3.1.8. *4-Methoxy-6-(methoxymethyl)-5-(oxiran-2-ylmethoxy)-1H-indole* (**8**) and *6-methoxy-4-(methoxymethyl)-5-(oxiran-2-ylmethoxy)-1H-indole* (**9**)

Indole derivatives **6 **and **7** were treated with (+/-)-epichlorohydrin in the presence of a stoichiometric quantity of base in dioxane and water according to the procedure described in the literature [1], to give the epoxides **8 **and **9 **with yields of 44% and 64%, respectively, as oils of chromatographic purity.

*Product* **8**: IR: 735.9, 1046.1, 1239.3, 1354.2, 1436.5, 1444.6, 1496.7, 1734.1, 2929.0, 3350.4 (NH) cm^-1^;^ 1^H-NMR (CDCl_3_): *δ* 2.71 and 2.87 (part AB of ABX system, *J*_AB_−4.9, 4.6 and 2.7, 2H, H-10), 3.38–3.41 (m, 1H, H-9), 3.44 (s, 3H, CH_2_OC*H*_3_), 3.98 (dd, *J*−11.2 and 6.3, 1H, H-8), 4.06 (s, 3H, OC*H*_3_), 4.27 (dd, *J*−11.2 and 3.2, 1H, H-8), 4.61 (AB system, *J* 11.6, 2H, C*H*_2_OCH_3_)_,_ 6.59–6.60 (m, 1H, H-3), 7.09 (s, 1H, H-7), 7.09–7.10 (m, 1H, H-2), 8.40 (brs, 1H, N*H*); ^13^C-NMR (CDCl_3_): *δ* 44.6 (*C*10), 50.8 (*C*9), 58.2 (CH_2_O*C*H_3_), 60.5 (O*C*H_3_), 70.6 (*C*H_2_OCH_3_), 74.9 (*C*8), 100.0 (*C*3), 106.5 (*C*7), 124.5 (*C*2), 121.4, 127.1, 134.0, 141.7, 144.9 (*C*-3a, 4, 5, 6, 7a); MS (EI, 70 eV), m/z [%]: 263 (48, M^+^), 206 (100), 191 (11), 176 (48), 147 (18), 132 (26), 118 (13); HR-MS for C_14_H_17_N_1_O_4_; calcd. 263.1158, found: 263.1165.

*Product* **9**: IR: 728.7, 846.8, 1086.0, 1143.9, 1199.2, 1301.1, 1459.5, 2924.5, 3369.9 (NH) cm^-1^; ^1^H-NMR (CDCl_3_): δ 2.70 and 2.86 (part AB of ABX system, *J*_AB_−5.0, 4.3 and 2.7, 2H, H-10), 3.38–3.41 (m, 1H, H-9), 3.42 (s, 3H, CH_2_OCH_3_), 3.80 (s, 3H, OCH_3_), 3.96 (dd, *J*−11.1 and 6.1, 1H, H-8), 4.18 (dd, *J*−11.1 and 3.5, 1H, H-8), 4.84 (AB system, *J* 10.8, 2H, CH_2_OCH_3_)_,_ 6.57–6.58 (m, 1H, H-3), 6.79 (s, 1H, H-7), 7.03 (dd, *J* 3.1 and 2.6, 1H, H-2), 8.47 (brs, 1H, NH); ^13^C-NMR (CDCl_3_): δ 44.7 (C10), 50.6 (C9), 56.0 (OCH_3_), 58.1 (CH_2_OCH_3_), 67.1 (CH_2_OCH_3_), 75.1 (C8), 95.0 (C7), 101.0 (C3), 123.5 (C2), 121.4, 122.0, 132.3 (C-3a, 4, 7a), 141.6 **(**C5), 149.5**(**C6); MS (EI, 70 eV), m/z [%]: 263 (M^+^, 100), 206 (98), 191 (15), 190 (23), 178 (51), 175 (32), 163 (22), 147 (35), 132 (30), 118 (34), 116 (34), 104 (20); HR-MS for C_14_H_17_NO_4_; calcd. 263.1158, found: 263.1148.

#### 3.1.9. *(2R,S)-1-(4-Methoxy-6-(methoxymethyl)-1H-indol-5-yloxy)-3-(2-(2-methoxyphenoxy)ethyl amino)propan-2-ol* (**10**) and *(2R,S)-1-(6-methoxy-4-(methoxymethyl)-1H-indol-5-yloxy)-3-(2-(2-methoxyphenoxy)ethylamino)propan-2-ol* (**11**)

Compounds **8** and **9** were converted into the final products **10** and **11 **using the procedure described in the literature [1]. They were obtained as foams with yields of 70% and 68%, respectively.

*Compound* **10**: IR: 1023.5, 1053.1, 1223.8, 1250.8, 1353.4, 1453.3, 1503.4, 3350.5 (NH and OH) cm^-1^; ^1^H-NMR (CDCl_3_): *δ* 2.17 (brs, 2H, O*H* and N*H*), 2.82–2.88 (m, 2H, H-3), 3.08 (t, *J* 5.5, 2H, H-4), 3.38 (s, 3H, OC*H*_3_), 3.83 (s, 3H, OC*H*_3_), 4.01 (dd, *J* 6.9 and 3.2, 1H, H-1), 4.04 (s, 3H, OC*H*_3_), 3.99–4.02 (m, 1H, H-2), 4.14 (t, *J* 5.5, 2H, H-5), 4.26 (dd, *J* 7.5 and 2.7, 1H, H-1), 4.58 (s, 2H, H-20)_,_ 6.59–6.60 (m, 1H, H-13), 6.87–6.96 (m, 4H, Ar*H*), 7.06 (s, 1H, H-18), 7.13–7.14 (m, 1H, H-12), 8.43 (brs, 1H, N*H*);^ 13^C-NMR (CDCl_3_): *δ* 48.8 (*C*4), 51.8 (*C*3), 55.8 (O*C*H_3_), 57.6 (O*C*H_3_), 60.5 (O*C*H_3_), 68.8 (*C*5), 69.5 (*C*2), 71.3 (*C*20), 77.1 (*C*1), 100.1 (*C*13), 107.5 (*C*18), 111.9, 114.1, 120.9, 121.5 (*C*8, 9, 10 and 11), 124.5 (*C*12), 126.2, 133.6 (*C*14 and 19), 121.7, 142.3, 144.7, 148.3, 149.7 (C6, 7, 15, 16 and 17); MS (EI, 70 eV), m/z [%]: 430 (6, M^+^), 415 (16), 354 (4), 261 (11), 224 (16), 207 (29), 175 (100), 132 (11), 56 (27); HR-MS for C_23_H_30_N_2_O_6_; calcd. 430.2104, found: 430.2106.

*Compound* **11**: IR: 731.5, 1250.5, 1453.8, 1503.8, 3309.5, 3350.7 (NH and OH) cm^-1^; ^1^H-NMR (CDCl_3_): δ 2.25 (brs, 2H, O*H* and N*H*), 2.84–2.86 (m, 2H, H-3), 3.07 (t, *J* 5.5, 2H, H-4), 3.40 (s, 3H, OC*H*_3_), 3.78 and 3.82 (2xs, 6H, 2xOC*H*_3_), 3.96 (dd, *J* 10.2 and 6.9, 1H, H-1), 4.05–4.10 (m, 1H, H-2), 4.12 (t, *J* 5.5, 2H, H-5), 4.16 (dd, *J* 10.2 and 3.0, 1H, H-1), 4.80 (s, 2H, H-20)_,_ 6.54–6.55 (m, 1H, H-13), 6.83 (s, 1H, H-18), 6.86–6.96 (m, 4H, Ar*H*), 7.08 (dd, *J* 3.0 and 2.5, 1H, H-12), 8.66 (brs, 1H, N*H*); ^13^C-NMR (CDCl_3_): δ 48.9 (*C*4), 51.8 (*C*3), 55.8 (*C*23), 56.0 (*C*22), 58.0 (*C*21), 67.3 (*C*20), 68.8 (*C*5), 69.5 (*C*2), 77,6 (*C*1), 95.0 (*C*18), 101.0 (*C*13), 111.9, 114.1, 120.9, 121.5 (*C*8, 9, 10, 11), 123.6 (*C*12), 121.4, 121.8, 132.0 **(***C*14, 15, 19), 142.0, 148.3 (*C*6, 16), 149.4, 149.7 (*C*7, 17); MS (EI, 70 eV), m/z [%]: 430 (11, M^+^), 415 (14, M^+^-CH_3_), 261 (14), 224 (10), 207 (38), 206 (23), 180 (21), 176 (26), 175 (100), 132 (9), 100 (10), 86 (12), 56 (26); HR-MS for C_23_H_30_N_2_O_6_; calcd. 430.2104, found: 430.2118.

### 3.2. Pharmacology

#### 3.2.1. Animals

The experiments were carried out on male Wistar rats (180–250 g) and male rabbits (2.5–3 kg). The animals were housed in constant temperature facilities exposed to 12:12 light-dark cycle, and maintained on a standard pellet diet, and tap water was given ad libitum. Control and experimental groups consisted of 6–8 animals each. The investigated compounds were administered intravenously at a constant volume of 1 mL∙kg^-1^. Control animals received the equivalent volume of solvent. All procedures were conducted according to guidelines of ICLAS (International Council on Laboratory Animals Science) and approved by The Local Ethic Committee on Animal Experimentation.

#### 3.2.2. Reference compound

Carvedilol was used as a reference drug.

#### 3.2.3. Statistical analysis

The data are expressed as the mean ± SEM. The statistical significance was calculated using a one-way ANOVA. Differences were considered as significant when P < 0.05.

#### 3.2.4. Adrenoceptor radioligand binding assay

The experiment was carried out on rat cerebral cortex. [^3^H]Prazosin (19.5 Ci mmol^-1^, an α_1_-adrenergic receptor), [^3^H]Clonidine (70.5 Ci mmol^-1^, an α_2_-adrenergic receptor), and [^3^H]CGP12177 (48 Ci mmol^-1^ an β_1_-adrenergic receptor) were used. The brains were homogenised in 20 vol of an ice-cold 50 mM Tris–HCl buffer (pH 7.6), and were centrifuged at 20.000 × *g* for 20 min (0–4 °C). The cell pellet was resuspended in the Tris–HCl buffer and centrifuged again. Radioligand binding assays were performed in plates (MultiScreen/Millipore). The final incubation mixture (final volume 300 μL) consisted of 240 μL of the membrane suspension, 30 μL of [^3^H]Prazosin (0.2 nM), [^3^H]Clonidine (2 nM) or [^3^H]CGP12177 (0.2 nM) solution, and 30 μL of the buffer containing seven to eight concentrations (10^–11^–10^–4^ M) of the tested compounds. For measuring the unspecific binding, phentolamine, 10 μM (in the case of [^3^H]Prazosin), 10μM (in the case of [^3^H]Clonidine), and propranolol – 1 μM (in the case of [^3^H]CGP12177) were applied. The incubation was terminated by rapid filtration over glass fibre filters (Whatman GF/C) using a vacuum manifold (Millipore). The filters were then washed twice with the assay buffer and placed in scintillation vials with a liquid scintillation cocktail. Radioactivity was measured in a WALLAC 1409 DSA liquid scintillation counter. All the assays were made in duplicate. The radioligand binding data were analyzed using an iterative curve-fitting routine (GraphPAD/Prism, Version 3.0, San Diego, CA, USA). K_i_ values were calculated from the Cheng and Prusoff equation [25].

#### 3.2.5. Effect on normal electrocardiogram (ECG)

Electrocardiographic measurement was carried out using the Ascard B5 Eco apparatus, standard lead II, and paper speed of 50 mm∙s^-1^. The tested compounds were administered intravenously in a dose of 1 mg∙kg^-1^. The ECG recording was carried out immediately before and 1, 5 and 15 min after administration of the tested compounds. The effect of the compounds on rat ECG recording was calculated according to Cambridge *et al*. [26].

#### 3.2.6. Prophylactic antiarrhythmic activity in a model of adrenaline-induced arrhythmia according to Szekeres and Papp [27]

Arrhythmia was evoked in thiopental (60 mg kg^-1^, ip) – anaesthetized rats by *iv* injection of adrenaline (20 μg kg^-1^). The tested compounds were administered intravenously 15 min or orally 60 min before adrenaline. The criterion of antiarrhythmic activity was the lack of premature beats and the inhibition of rhythm disturbances in comparison with the control group (ventricular bradycardia, atrioventricular block, ventricular tachycardia or ventricular fibrillation). The cardiac rhythm disturbances were recorded for 15 min after adrenaline injection. ECGs were analyzed according to the guidelines of the Lambeth Convention [28] on ventricular premature beats (VBs), bigeminy, salvos (less than four successive VBs), ventricular tachycardia (VT, four or more successive VBs), and ventricular fibrillation (VF).

#### 3.2.7. Influence on blood pressure in rats

Male Wistar normotensive rats were anesthetized with thiopental (50–75 mg kg^-1^, *ip*). The right carotid artery was cannulated with a polyethylene tube, filled with heparin in saline to facilitate pressure measurement using the Datamax apparatus (Columbus Instruments). The studied compounds were injected in a single dose of 1.0–0.003 mg kg^-1^ into the caudal vein or given *per os* in a single dose of 1.0–0.03 mg kg^-1^ after a 5 min stabilization period at a volume equivalent to 1 mL kg^-1^.

#### 3.2.8. Influence on isolated rabbit ileum

The influence on isolated rabbit ileum of the investigated compounds on the smooth muscle was investigated on the rabbit small intestine, according to the Magnus’ method [29] modified and described by Orisadipe *et al*. [30] The white rabbits were sacrificed by cervical dislocation and the small intestine was immediately removed, and cut into strips about 3–4 cm long. The isolated strips were incubated in Krebs solution at 37 °C and aerated with carbogen in special laboratory dishes. The isolated strip of the intestine was placed in a test glass tube with the Krebs solution constantly aerated by carbogen. After 1 hour incubation period, during which the physiological saline solution was changed every 15 min, the influence of the investigated compounds on spontaneous contractions of the rabbit ileum was evaluated. The contraction of the intestine was recorded on a TZ-4100 line recorder *via* isometric Harvard transducer, at the muscle load of 1 g. The influence of every single dose was recorded for 5 min.

## 4. Conclusions

The results of this study confirmed that the compounds (2*R,S*)-1-(6-methoxy-4-(methoxymethyl)-1*H*-indol-5-yloxy)-3-(2-(2-methoxyphenoxy)ethylamino)propan-2-ol (**10**) and (2*R,S*)-1-(4-methoxy-6-(methoxymethyl)-1*H*-indol-5-yloxy)-3-(2-(2-methoxyphenoxy)ethylamino)propan-2-ol (**11**) possess α-adrenolytic and weak spasmolytic activities. The introduction of the aminopropanol moiety into position 5- instead of position 4- of the indole ring of the compound **(*R,S*)-9 **suppresses β-adrenolytic activity. The results suggest that the antiarrhythmic and hypotensive effects of the tested compounds are related with their adrenolytic properties, but the pharmacological effects of the tested compounds were qualitatively weaker than those of carvedilol and **(*R,S*)-9**.
